# The Impact of an Intensive Care Unit Antimicrobial Stewardship Program on the Appropriateness of Meropenem Prescribing at a Tertiary Hospital: A Retrospective Quasi-Experimental Study

**DOI:** 10.3390/antibiotics15101013

**Published:** 2026-10-13

**Authors:** Lina I. Alnajjar, Taif A. Alahmari, Shahad A. Almutairi, Rawan A. Alniwaider, Mercy Joseph, Abdullah K. Alruwaili, Alaa A. Alhubaishi, Ahlam Alghamdi, Anwar M. Alnakhli, Abdulrahman S. Alissa, Amani A. Bahdailah, Mohammed A. Alraey, Isra Z. Farooqi, Asem A. Allam, Reem Binsuwaidan

**Affiliations:** 1 Department of Pharmacy Practice, College of Pharmacy, Princess Nourah bint Abdulrahman University, P.O. Box 84428, Riyadh 11671, Saudi Arabia; lialnajjar@pnu.edu.sa (L.I.A.); aaalhubaishi@pnu.edu.sa (A.A.A.); ahaalghamdi@pnu.edu.sa (A.A.); 2 College of Pharmacy, Princess Nourah bint Abdulrahman University, P.O. Box 84428, Riyadh 11671, Saudi Arabia; taifalahmari6@hotmail.com (T.A.A.); shhdaalmutairi@gmil.com (S.A.A.); ralniwaider@gmail.com (R.A.A.); 3 Department of Infection Prevention and Control, King Abdullah bin Abdulaziz University Hospital, Princess Nourah bint Abdulrahman University, P.O. Box 84428, Riyadh 11671, Saudi Arabia; mjoseph@kaauh.edu.sa; 4 Department of Internal Medicine, King Abdullah bin Abdulaziz University Hospital, Princess Nourah bint Abdulrahman University, P.O. Box 84428, Riyadh 11671, Saudi Arabia; dr.abdullah0012@gmail.com; 5 Department of Pharmaceutical Sciences, College of Pharmacy, Princess Nourah bint Abdulrahman University, P.O. Box 84428, Riyadh 11671, Saudi Arabia; amalnklee@pnu.edu.sa; 6 Department of Pharmaceutical Care Services, King Abdullah bin Abdulaziz University Hospital, Princess Nourah bint Abdulrahman University, P.O. Box 84428, Riyadh 11671, Saudi Arabia; asalissa@kaauh.edu.sa (A.S.A.); aabahdailah@kaauh.edu.sa (A.A.B.); 7 Department of Infectious Diseases, King Abdullah bin Abdulaziz University Hospital, Princess Nourah bint Abdulrahman University, P.O. Box 84428, Riyadh 11671, Saudi Arabia; maalraey@kaauh.edu.sa (M.A.A.); izfarooqi@kaauh.edu.sa (I.Z.F.); aaallam@kaauh.edu.sa (A.A.A.)

**Keywords:** antimicrobial stewardship, intensive care unit, meropenem, carbapenems, antimicrobial resistance

## Abstract

**Background:** Carbapenem-resistant Enterobacteriaceae (CRE) prevalence has been increasing in Saudi Arabia, posing a significant public health concern. Antimicrobial stewardship programs (ASPs) play an important role in reducing resistance by ensuring appropriate antibiotic use. Our study aimed to assess the appropriateness of meropenem prescriptions pre-ASP and post-ASP implementation in the Intensive Care Unit (ICU) ward. **Method:** This is a retrospective quasi-experimental pre–post study conducted over 3 years, from 2020 to 2023 at King Abdullah bin Abdulaziz University Hospital (KAAUH). The study included adult ICU patients who received meropenem therapy. The primary objective was to evaluate the appropriateness of meropenem prescriptions in the ICU before and after ASP implementation. The secondary objectives were to identify reasons for inappropriate prescribing and evaluate the interventions implemented to address inappropriate meropenem use. **Result:** A total of 113 patients were included: 34 in 2020, 41 in 2022, and 38 in 2023. Appropriate meropenem prescribing increased significantly from 23.5% in 2020 to 26.8% in 2022 and 57.9% in 2023 (*p* = 0.003), while inappropriate prescribing decreased from 76.5% to 73.2% and 42.1%, respectively. Treatment duration also differed significantly across study periods (*p* = 0.002), with courses of 7–10 days increasing from 24.2% in 2020 to 53.7% in 2022 and 52.6% in 2023, while courses exceeding 10 days decreased from 51.5% to 12.2% and 18.4%, respectively. Among inappropriate prescriptions, an inappropriate antimicrobial spectrum was the predominant reason (81.9%), with no inappropriate dosing identified. Documented stewardship interventions differed significantly across periods (*p* < 0.001): de-escalation increased from 0% in 2020 to 43.3% in 2022 and 50.0% in 2023, while prescriptions completed without documented intervention decreased from 69.2% to 23.3% and 18.8%, respectively. **Conclusion:** ASP implementation was associated with a significant improvement in the appropriateness of meropenem prescribing and a shift toward shorter treatment durations among adult ICU patients. Nevertheless, inappropriate prescribing remained substantial in the second post-implementation year, emphasizing the need for continued antimicrobial stewardship interventions and ongoing monitoring of carbapenem use.

## 1. Introduction

Meropenem is a broad-spectrum carbapenem antibiotic with activity against a wide range of Gram-negative and Gram-positive organisms, as well as anaerobic pathogens. It is frequently used for severe infections in critically ill patients, particularly when multidrug-resistant Gram-negative organisms are suspected or confirmed. However, its broad spectrum and importance in treating serious resistant infections make meropenem a valuable antimicrobial that requires judicious use to preserve its effectiveness [1].

Carbapenem-resistant Gram-negative bacteria, including carbapenem-resistant Enterobacterales (CRE), Pseudomonas aeruginosa, and Acinetobacter baumannii, represent an important global antimicrobial resistance challenge. Carbapenem resistance may result from several mechanisms, including carbapenemase production, reduced membrane permeability, increased efflux activity, and combinations of these mechanisms. The global dissemination of carbapenem-resistant Enterobacterales has been associated with diverse resistance mechanisms and presents major therapeutic challenges because treatment options for affected patients are frequently limited [2].

The burden of carbapenem resistance is of particular concern in healthcare settings because carbapenems are often reserved for the treatment of severe infections caused by multidrug-resistant Gram-negative organisms. Inappropriate or unnecessary exposure to these agents may increase antimicrobial selection pressure and contribute to the emergence and dissemination of resistant organisms. Consequently, optimizing carbapenem prescribing is an important component of antimicrobial resistance prevention and antimicrobial stewardship strategies [1,2].

Carbapenem resistance has also become an important concern in Saudi Arabia. A systematic review and meta-analysis reported a substantial prevalence of carbapenem-resistant Enterobacterales in Saudi Arabia, highlighting the continuing national burden of carbapenem resistance and the need for effective strategies to optimize antimicrobial use [3]. Furthermore, a multicenter study of patients with confirmed CRE Escherichia coli or Klebsiella pneumoniae infections in Saudi Arabia reported considerable morbidity and mortality, with approximately 30% of patients dying within 30 days. CRE bacteremia was independently associated with increased mortality, demonstrating the clinical consequences of these resistant infections [4].

The epidemiology of carbapenem resistance in Saudi Arabia is heterogeneous, with regional differences in antimicrobial susceptibility patterns and resistance mechanisms. OXA-48-type carbapenemases have been reported among carbapenem-resistant Enterobacterales in Saudi healthcare settings, although other carbapenemase mechanisms have also been identified. This diversity in resistance mechanisms can complicate antimicrobial selection and reinforces the importance of appropriate antimicrobial therapy and infection control measures [5].

Antimicrobial stewardship programs (ASPs) are an important strategy for optimizing antimicrobial therapy. ASPs aim to ensure appropriate antimicrobial selection, dosing, route of administration, and duration while minimizing adverse effects and limiting the development and spread of antimicrobial resistance. Stewardship activities may include prospective audit and feedback, antimicrobial preauthorization, development of treatment guidelines, education, and multidisciplinary review of antimicrobial therapy. Evidence from Saudi hospitals indicates that ASP implementation can improve antimicrobial prescribing, although the availability and maturity of stewardship activities vary between institutions [6].

The intensive care unit (ICU) is a particularly important setting for antimicrobial stewardship because critically ill patients have a high burden of suspected or confirmed infection and are frequently exposed to broad-spectrum antimicrobials. Critically ill patients may also have altered pharmacokinetics and pharmacodynamics, complex comorbidities, and an increased risk of infection with multidrug-resistant organisms. These factors make antimicrobial optimization particularly challenging in the ICU [7,8].

In addition, the urgency of initiating empiric antimicrobial therapy in critically ill patients may result in continued broad-spectrum treatment even after microbiological and clinical information becomes available. Contemporary ICU stewardship therefore emphasizes timely initiation of appropriate empiric therapy followed by systematic reassessment, optimization of antimicrobial exposure, de-escalation when appropriate, and avoidance of unnecessarily prolonged treatment. Multidisciplinary collaboration between intensivists, infectious diseases physicians, pharmacists, microbiologists, nurses, and other healthcare professionals is an important component of effective ICU stewardship [7,8,9].

Although antimicrobial stewardship has become increasingly established internationally and in Saudi Arabia, evidence specifically evaluating the appropriateness of meropenem prescribing following implementation of an ICU-focused stewardship program in a Saudi tertiary hospital remains limited. In particular, evaluating prescribing according to specific components such as antimicrobial spectrum, dose, and treatment duration can help identify the aspects of meropenem use that remain vulnerable to inappropriate prescribing despite implementation of a stewardship program [6,7,9].

Therefore, this study aimed to assess the appropriateness of meropenem prescribing before and after implementation of an ICU-focused ASP in the adult ICU at King Abdullah bin Abdulaziz University Hospital (KAAUH). This study also aimed to identify the principal reasons for inappropriate meropenem prescribing and characterize the stewardship interventions implemented in response to inappropriate prescriptions.

## 2. Methods

### 2.1. Study Design and Setting

This retrospective quasi-experimental pre–post study was conducted at King Abdullah bin Abdulaziz University Hospital from 2020 to 2023.

The Antimicrobial Stewardship Program (ASP) was implemented in 2021. To allow for an implementation and stabilization period, 2021 was considered a transition year and was excluded from the analysis. Accordingly, patients treated in 2020 were considered the pre-ASP group, whereas patients treated during 2022 and 2023 were considered the post-ASP groups.

The ASP team consisted of infectious diseases (IDs) physicians, clinical pharmacists, clinical microbiologists, infection prevention and control specialists, and nurses. An adult ICU ASP subcommittee was established in 2021 and included an adult ID physician, ID clinical pharmacist, ICU physician, ICU clinical pharmacist, and ICU nurse.

The ICU ASP interventions included the implementation of empiric antimicrobial treatment guidelines, restriction of broad-spectrum antimicrobial agents through mandatory preauthorization by an ID physician or ID clinical pharmacist, prospective audit and feedback, weekly multidisciplinary ICU meetings, and educational sessions.

### 2.2. Study Sample and Time Frame

All adult patients (>18 years) who were hospitalized in Intensive Care Units and were prescribed meropenem for any indication from January 2020 until December 2023. Patients admitted to other wards or received other than meropenem were excluded from the study. All patients from the transition year 2021 were excluded.

### 2.3. Data Collection

Patients were identified using the hospital’s computerized information system. Patient data were collected by a trained researcher and included demographic characteristics (age and gender), hospital ward, indication for meropenem therapy, and basis of prescription (empiric or culture-guided), the dose of administered meropenem, the duration of the meropenem treatment and its correctness, and the type of intervention.

### 2.4. Assessment of Meropenem Appropriateness

The appropriateness of meropenem prescribing was assessed according to three predefined criteria: dose, treatment duration, and antimicrobial spectrum.

Antimicrobial spectrum was considered appropriate when the isolated organism was susceptible to meropenem and resistant to other narrower-spectrum antimicrobial agents, based on the available microbiological susceptibility results. Dose and treatment duration were assessed according to the applicable clinical and institutional recommendations.

A prescription was classified as appropriate when all three criteria were appropriate and as inappropriate when at least one criterion was inappropriate.

Documented antimicrobial stewardship interventions were also assessed and categorized as escalation, de-escalation, discontinuation, or no change. Escalation was defined as broadening or intensifying antimicrobial therapy based on the available microbiological culture and susceptibility results. De-escalation was defined as narrowing antimicrobial therapy based on microbiological results, clinical response, or both. Discontinuation referred to stopping meropenem therapy, whereas no change indicated continuation of the existing antimicrobial regimen without a documented stewardship intervention.

### 2.5. Study Outcome

The primary outcome was the appropriateness of meropenem prescribing based on antimicrobial spectrum, dose, and treatment duration. Secondary outcomes included the reasons for inappropriate prescribing, duration of meropenem therapy, and documented stewardship interventions, including de-escalation and discontinuation.

### 2.6. Statistical Analysis

Categorical data were summarized using counts and percentages, while continuous data non-normally distributed were summarized using the median and interquartile range (IQR).

The normality of continuous data was assessed both visually, through Normal Q-Q plots and histograms, and statistically, using the Shapiro–Wilk test. Non-normally distributed continuous variables were compared across the three study periods using the Kruskal–Wallis test, a non-parametric method.

To compare categorical data, a chi-square (X2) test was conducted to assess associations. Fisher’s exact test was applied where appropriate, when expected cell counts were less than 5.

Statistical significance was set at *p*-value of <0.05. Statistical analyses were performed using IBM SPSS Statistics version 29.

## 3. Results

A total of 113 eligible adult ICU patients who received meropenem were included in the study. The pre-ASP group consisted of 34 patients treated in 2020. Following exclusion of the 2021 transition period, 79 patients were included in the post-ASP period: 41 patients in 2022 and 38 patients in 2023.

### 3.1. Baseline Characteristics Between Pre-ASP and Post-ASP Groups

The baseline characteristics of the study sample, stratified by study period, are presented in Table 1. The median age of all patients was 66 years (IQR: 48–83) and was increased significantly across the study periods (*p* = 0.001), with the highest median age observed in 2023.

Females represented 56.6% of the total study sample, with no statistically significant difference in sex distribution between the pre-ASP and post-ASP groups (*p* = 0.32).

Meropenem was prescribed empirically in 46.9% of all patients and was used as escalation therapy in 46%. Compared with the pre-ASP period, the distribution of therapy type differed significantly after ASP implementation (*p* = 0.004), with empirical therapy increasing in the first post-ASP year and escalation therapy decreasing during that same period. Sepsis and hypoxemia remained uncommon across all study periods.

Among patients with documented microbiological cultures, sputum was the most frequently reported culture source, followed by urine, wound, and blood specimens. No statistically significant differences were observed between the pre-ASP and post-ASP groups in the distribution of culture sources.

### 3.2. Appropriateness of Meropenem Prescriptions Before and After ASP Implementation

The proportion of appropriate meropenem prescriptions increased significantly across the study periods, from 23.5% in 2020 and 26.8% in 2022 to 57.9% in 2023 (*p* = 0.003) (Table 2).

Treatment duration also differed significantly across the study periods (*p* = 0.02). The proportion of patients receiving meropenem for 7–10 days increased from 24.2% in 2020 to 53.7% in 2022 and 52.6% in 2023. In contrast, treatment durations exceeding 10 days decreased from 51.5% in 2020 to 12.2% in 2022 and 18.4% in 2023.

### 3.3. Reason for Inappropriate Meropenem Prescription

No cases of inappropriate meropenem dosing were identified in either the pre-ASP or post-ASP groups. An inappropriate antimicrobial spectrum was the most common reason for inappropriate prescribing, accounting for 81.9% of cases overall, while duration-related inappropriateness accounted for 18.1% of cases overall and with no statistical significant difference between the pre-ASP and post-ASP groups (*p* = 0.8).

Regarding stewardship interventions, the distribution of actions taken for inappropriate prescriptions differed significantly across the study periods (*p* < 0.001). Before ASP implementation, most inappropriate prescriptions were completed without action (69.2%). After ASP implementation, this decreased to 23.3% in 2022 and 18.8% in 2023, while de-escalation increased from 0.0% in 2020 to 43.3% in 2022 and 50.0% in 2023. Discontinuation of treatment remained relatively similar across the study periods. (Table 3).

## 4. Discussion

This study evaluated the appropriateness of meropenem prescribing before and after implementation of an ICU-focused antimicrobial stewardship program at a tertiary teaching hospital in Saudi Arabia. The principal finding was a significant improvement in prescribing appropriateness over the study period. Appropriate meropenem prescribing increased from 23.5% in 2020 to 26.8% in 2022 and 57.9% in 2023, while inappropriate prescribing decreased from 76.5% to 42.1%. In parallel, treatment courses exceeding 10 days decreased substantially post ASP implementation, and documented stewardship interventions, particularly de-escalation and discontinuation, became more frequent. Taken together, these findings suggest that the ICU-focused ASP was associated with progressive improvement in several components of meropenem use; however, the pre–post design does not allow for definitive causal attribution of these changes to the ASP alone.

The improvement in meropenem prescribing observed in our study is consistent with evidence from carbapenem-focused stewardship interventions. Yoo et al. reported that a prolonged carbapenem-focused antimicrobial stewardship intervention was associated with reduced antimicrobial consumption and improvements in antimicrobial prescribing [10]. Similarly, García-Rodríguez et al. demonstrated improvements in carbapenem prescribing following implementation of a long-term carbapenem stewardship program, suggesting that sustained stewardship activity can influence carbapenem utilization over time [11]. In another healthcare setting, Seah et al. reported that implementation of a carbapenem antimicrobial stewardship program was associated with favorable patient outcomes, providing further support for targeted stewardship approaches to optimize carbapenem use [12]. The progressive improvement observed in the present study, particularly the larger increase in appropriateness during the second post-implementation year, is also consistent with the concept that stewardship effects may require sustained implementation, repeated feedback, and continued engagement with prescribers rather than being limited to the initial implementation period [10,11].

The improvement observed in the present study may be attributable to the combination of interventions implemented through the ICU-ASP, including empiric antimicrobial guidelines, mandatory preauthorization, prospective audit and feedback, multidisciplinary meetings, and educational activities. These interventions address different determinants of antimicrobial prescribing and may therefore have complementary effects. The multifaceted nature of these interventions is important because antimicrobial prescribing in the ICU is influenced by clinical severity, microbiological uncertainty, prescriber experience, and the need to balance timely treatment with avoidance of unnecessary antimicrobial exposure. The present findings therefore support the use of a structured, multidisciplinary approach rather than reliance on a single stewardship intervention [7,9].

Despite the overall improvement, inappropriate prescribing remained substantial in 2023, with 42.1% of meropenem prescriptions classified as inappropriate. Inappropriate antimicrobial spectrum was the predominant reason, accounting for 81.9% of inappropriate prescriptions. This finding suggests that although the ICU-ASP was associated with improved overall prescribing, optimizing the spectrum of antimicrobial therapy remains an important area for further intervention. The predominance of spectrum-related inappropriateness is particularly relevant because meropenem may be initiated appropriately as empiric therapy in critically ill patients but may become unnecessarily broad once culture results, susceptibility data, or clinical response are available. Continued review of the indication for meropenem, microbiological results, susceptibility patterns, and clinical response may help identify opportunities to narrow therapy when broad-spectrum coverage is no longer justified. This finding also indicates that future stewardship efforts should not focus solely on whether meropenem is indicated, but also on whether its spectrum remains justified throughout the course of treatment [7,9,13].

The reduction in prolonged treatment courses represents another important finding. The proportion of patients receiving meropenem for more than 10 days decreased from 51.5% in the pre-ASP period to 12.2% in 2022 and 18.4% in 2023. This pattern is consistent with the broader stewardship principle of minimizing unnecessary antimicrobial exposure. In the ICU, treatment duration should be reassessed according to the suspected or confirmed source of infection, microbiological findings, clinical response, and adequacy of source control rather than continued automatically once therapy has been initiated [13,14]. Although the proportion receiving more than 10 days increased slightly from 2022 to 2023, it remained substantially below the pre-ASP level, suggesting that the reduction in prolonged therapy was largely maintained during the observed post-implementation period.

De-escalation was documented in 50.0% of inappropriate prescriptions in 2023, compared with 43.3% in 2022 and none in 2020. These findings suggest that establishment of the ICU-focused ASP was associated not only with improved prescribing appropriateness but also with greater active intervention when meropenem therapy was considered inappropriate. De-escalation is an important component of antimicrobial stewardship in critically ill patients because it allows initial empiric therapy to be modified according to subsequent clinical and microbiological information [13,14]. The absence of documented de-escalation in the pre-ASP group and its subsequent occurrence post implementation may indicate a change in the clinical management process, with greater emphasis on reassessment of broad-spectrum therapy. However, because intervention documentation was not necessarily complete, these findings should be interpreted as documented stewardship activity rather than a direct measure of all ASP activity.

The increase in documented stewardship interventions may also reflect improved communication and collaboration between the stewardship team and treating clinicians. Alba Fernandez et al. evaluated a meropenem stewardship program and demonstrated the importance of acceptance of stewardship recommendations, highlighting the role of collaboration between stewardship teams and prescribers in optimizing antimicrobial treatment [15]. In the present study, the greater frequency of documented de-escalation and discontinuation post implementation of the ICU-ASP may therefore reflect increased engagement of the stewardship team in day-to-day antimicrobial decision-making. This is particularly relevant in the ICU, where antimicrobial decisions often need to be revisited rapidly as clinical and microbiological information evolves [7,9].

Our findings are also consistent with evidence from Saudi Arabia supporting the effectiveness of antimicrobial stewardship programs. Al-Omari et al. reported a reduction in inappropriate antibiotic use following implementation of an ASP across four tertiary hospitals in Saudi Arabia [16]. Previous work specifically evaluating meropenem prescribing in the region has also demonstrated substantial inappropriate use. Al-Hadithi et al. reported that 51% of meropenem prescriptions were inappropriate in a tertiary care hospital in Oman, highlighting the challenges associated with optimizing use of this broad-spectrum agent [17]. Similarly, Alsaleh et al. evaluated carbapenem and piperacillin-tazobactam prescribing in a tertiary hospital in Saudi Arabia and identified opportunities to improve antimicrobial appropriateness [18]. Together, these findings support the need for sustained stewardship efforts targeting broad-spectrum antimicrobial use in tertiary healthcare settings. The relatively high level of inappropriate prescribing observed before ASP implementation in our study is therefore consistent with the broader regional challenge of optimizing broad-spectrum antimicrobial use, while the subsequent improvement provides further support for structured stewardship approaches in tertiary-care settings [16,17,18].

The persistent level of inappropriate spectrum in our study also indicates that improvement in overall appropriateness does not necessarily translate into complete optimization of antimicrobial selection. Future interventions could therefore focus more specifically on culture-guided de-escalation, regular review of antimicrobial indications, documentation of treatment plans, and targeted feedback to prescribers regarding spectrum selection. Such approaches may be particularly valuable in the ICU, where empirical broad-spectrum therapy is often necessary initially but should be reassessed as additional clinical and microbiological information becomes available. The fact that spectrum accounted for more than four-fifths of inappropriate prescriptions in this cohort provides a specific target for future quality improvement interventions and suggests that monitoring spectrum appropriateness separately from overall appropriateness may be useful for evaluating the impact of the ICU-ASP [7,9,13].

This study has several limitations. First, this was a retrospective single-center study, which may limit the generalizability of the findings to other hospitals or ICUs. Second, the sample size was relatively small, which may have limited the ability to detect differences in some secondary outcomes. Third, documentation of stewardship interventions was not always consistent; therefore, the observed frequency of interventions may underestimate the actual activity of the ASP. Additionally, the study focused specifically on meropenem prescribing and did not assess the utilization of other carbapenems; therefore, potential substitution with other carbapenem agents could not be evaluated.

Despite these limitations, this study provides useful evidence regarding the effect of an ICU-focused ASP on meropenem prescribing in a Saudi tertiary-care setting. The improvement in appropriateness, reduction in prolonged treatment courses, and increase in documented stewardship interventions suggest that a structured ICU-based approach can meaningfully influence the use of a high-value broad-spectrum antimicrobial. However, the persistence of spectrum-related inappropriate prescribing demonstrates that stewardship should be viewed as an ongoing process requiring continuous monitoring, feedback, education, and multidisciplinary collaboration. The findings also suggest that the benefits of stewardship may extend beyond simply reducing antimicrobial use, by promoting more individualized decisions regarding spectrum, duration, and continuation or discontinuation of therapy. Continued monitoring will be important to determine whether the improvements observed in this study can be sustained and further improved over time.

## 5. Conclusions

Implementation of the ICU-focused ASP was associated with improved meropenem prescribing appropriateness, reduced prolonged treatment courses, and increased documented stewardship interventions, particularly de-escalation. However, an inappropriate antimicrobial spectrum remained the main reason for inappropriate prescribing. Continued multidisciplinary stewardship interventions, prospective audit and feedback, and monitoring of meropenem use are warranted to further optimize carbapenem prescribing in the ICU.

## Figures and Tables

**Table 1:** Comparison of baseline characteristics between the patients in pre-ASP and post-ASP implementation groups.

Variable	Pre-ASP Implementation	Post-ASP Implementation		*p*-Value
	2020 (*n* = 34)	2022 (*n* = 41)	2023 (*n* = 38)	
Age (median, IQR) years	57 (34.75–70.50)	61 (48–79.50)	83 (67.50–85)	0.001
Gender, *n* (%)				
Male	16 (47.1%)	14 (34.1%)	19 (50%)	0.32
Female	18 (52.9%)	27 (65.9%)	19 (50%)	
Type of therapy, *n* (%)				
Empirically	12 (36.4%)	26 (66.7%)	15 (39.5%)	0.004
Escalation	21 (63.6%)	9 (23.1%)	22 (57.9%)	
Sepsis	0 (0%)	3 (7.7%)	1 (2.6%)	
Hypoxemia	0 (0%)	1 (2.6%)	0 (0%)	
Culture site, *n* (%)				
Blood	3 (10.7%)	1 (3.6%)	4 (13.3%)	0.27
Sputum	21 (75%)	17 (60.7%)	15 (50%)	
Urine	2 (7.1%)	7 (25%)	5 (16.7%)	
Wound	2 (7.1%)	3 (10.7%)	6 (20%)	

**Table 2:** Appropriateness of meropenem prescription in pre-ASP and post-ASP implementation groups.

Variable	Pre-ASP Implementation	Post-ASP Implementation		*p*-Value
	2020 (*n* = 34)	2022 (*n* = 41)	2023 (*n* = 38)	
Appropriateness, *n* (%)				
Appropriate	8 (23.5%)	11 (26.8%)	22 (57.9%)	0.003
Inappropriate	26 (76.5%)	30 (73.2%)	16 (42.1%)	
Days of therapy, *n* (%)				
1–3	1 (3%)	8 (19.5%)	7 (18.4%)	0.002
4–6	7 (21.2%)	6 (14.6%)	4 (10.5%)	
7–10	8 (24.2%)	22 (53.7%)	20 (52.6%)	
>10	17 (51.5%)	5 (12.2%)	7 (18.4%)	

**Table 3:** Inappropriateness of meropenem prescription (*n* = 72).

Variable	Pre-ASP Implementation	Post-ASP Implementation		*p*-Value
	2020 (*n* = 26)	2022 (*n* = 30)	2023 (*n* = 16)	
Reasons for Inappropriateness, *n* (%)				
Spectrum	20 (76.9%)	25 (83.3%)	14 (87.5%)	0.8
Duration	6 (23.1%)	5 (16.7%)	2 (12.5%)	
Implemented action of inappropriateness, *n* (%)				
No action was taken (completed treatment)	18 (69.2%)	7 (23.3%)	3 (18.8%)	<0.001
Discontinuation of treatment	8 (30.8%)	10 (33.3%)	5 (31.3%)	
De-escalation	0 (0.0%)	13 (43.3%)	8 (50%)	

## Data Availability

The data presented in this study are available on request from the corresponding author. The data are not publicly available due to patients’ information privacy.
